# Cellular computation and cognition

**DOI:** 10.3389/fncom.2023.1107876

**Published:** 2023-11-23

**Authors:** W. Tecumseh Fitch

**Affiliations:** Faculty of Life Sciences and Vienna Cognitive Science Hub, University of Vienna, Vienna, Austria

**Keywords:** computational neuroscience, dendrites, cognition, dendritic computing, neural network models, cellular computing

## Abstract

Contemporary neural network models often overlook a central biological fact about neural processing: that single neurons are themselves complex, semi-autonomous computing systems. Both the information processing and information storage abilities of actual biological neurons vastly exceed the simple weighted sum of synaptic inputs computed by the “units” in standard neural network models. Neurons are eukaryotic cells that store information not only in synapses, but also in their dendritic structure and connectivity, as well as genetic “marking” in the epigenome of each individual cell. Each neuron computes a complex nonlinear function of its inputs, roughly equivalent in processing capacity to an entire 1990s-era neural network model. Furthermore, individual cells provide the biological interface between gene expression, ongoing neural processing, and stored long-term memory traces. Neurons in all organisms have these properties, which are thus relevant to all of neuroscience and cognitive biology. Single-cell computation may also play a particular role in explaining some unusual features of human cognition. The recognition of the centrality of cellular computation to “natural computation” in brains, and of the constraints it imposes upon brain evolution, thus has important implications for the evolution of cognition, and how we study it.

## Introduction

The inception of modern computer science can be traced directly to three giants: Alan Turing, John von Neumann, and Claude Shannon. Both Turing and von Neumann had plenty to say about the brain, and how their respective notions of computation might somehow be mapped onto neural tissue (Turing, 1950; von Neumann, 1958), and although it seems likely that Shannon was aware of these ideas, he did not publish on this topic himself. Nonetheless, Shannon made crucial contributions to the implementation of computation on machines (Shannon, 1938), as well as single-handedly creating information and coding theory (Shannon, 1948), and these contributions remain fundamental to contemporary computational neuroscience. In this paper I will attempt to apply Shannon’s computational and informational tools to a fundamental question in brain research: how do single neurons contribute to cognition? I will argue that individual cells play fundamental roles in both neural computation and information storage (memory), roles vastly exceeding those envisioned in “standard” contemporary neural network models. If correct, this argument has important implications for cognitive neuroscience, particularly regarding the evolution of cognition in animals.

Debates about the computational role of individual neurons go back more than a century, to the origins of neuroscience (Shepherd, 1991; Finger, 2000). A central debate in early neuroscience pitted the network or “reticular” theorists like Camillo Golgi, who believed that the cortex constituted a vast web of continuously interconnected cytoplasm, against those who saw the brain as composed of individual cells termed “neurons.” The champion of the neuron-based viewpoint was Santiago Ramon y Cajal, whose remarkable images of neurons, stained with Golgi’s method, provided increasing evidence that the brain is constructed of independent cells, connected not by fusion but by contact at synapses (Shepherd, 1991). The theory was consistent with the established notion that most other body tissues in plants and animals are made up of separate cells – “cell theory” - and is now enshrined as the universally accepted “neuron doctrine” (Bear et al., 2001). By the time Golgi and Cajal shared the Nobel Prize in 1906, most biologists had accepted the neuron doctrine, although Golgi himself sided with the reticular concept until his death.

Cajal argued that neurons are the fundamental units of neural processing. He recognized that neurons are dynamic entities, changing their connections to other cells and changing their own form, and believed that such changes played a critical role in learning and development. Cajal’s student Lorente de No, an early neurophysiologist, further developed these ideas, arguing that electrical activity (today we would say “information”) mostly flowed one way in a cell, from dendrites to the cell body and down the axon. Today, with a few tweaks, these insights have become standard textbook neuroscience.

It is thus ironic that, when Turing, Shannon and von Neumann were establishing computer science, a rather different conception of neural computation was in vogue. The underlying theory in so-called “neural network models,” extending from Donald Hebb’s theoretical work in the 1940s, through the earliest perceptron models of the 1960s, through connectionist models in the 1990s, to today’s “deep” neural nets, dispenses with the complex and beautiful dendritic and axonal trees that Cajal spent his life meticulously documenting. The “units” in these artificial neural networks (ANNs) are simple summators only loosely modeled on neurons, and connected by “weights” modeled on synapses. They are in these respects consistent with the neuron doctrine. However, the only computation these units perform is to linearly sum their weighted inputs and apply a nonlinear threshold to the result (see below). The information content of such units is stored entirely in their synaptic weights, and the computation performed depends on the structure of the network that encompasses them, not any property of the unit. In these and other ways, the units upon which neural networks are based deviate sharply from real biological neurons.

If alive today, Cajal might well ask why the complex and changing three-dimensional forms of his beloved neurons have been reduced to a simple spherical blob, and any potential information content or computation instantiated in that complex form has disappeared. Indeed, he might see the modern networks used in machine learning and AI today as a conceptual step backwards, toward the reticular model of Golgi, rather than being “neural” in any real sense. Fortunately, practicing neuroscientists never stopped studying cells, and we now know that real neurons play a much more powerful role, in terms of both information storage and computation, than the units of an ANN. As I will detail below, each neuron is a complex computer in its own right, at multiple levels, and this has serious implications for our theoretical understanding of brains and cognition. In Shannon’s terms, both the computational hardware (Shannon, 1938) and information content (Shannon, 1948) of the brain need to be grounded at the cellular level of individual neurons if we are to understand computation and memory in real nervous systems, and their evolution.

In this paper I argue that an adequate understanding of neural computation must incorporate what I will term “cellular computing,” a term encompassing not only “standard models” of synapses and spikes, but also nano-scale biochemical and genetic information processing, micro-scale morphology of neurons, and meso-scale cell–cell interactions (including the connectome). Some principles underlying each of these distinct levels of biophysical information processing have been known for decades, e.g., that neurons are slow and sloppy compared to transistors, or that brains are massively parallel computational systems (Rummelhart and McClelland, 1986). Other principles have only recently become clear, such as understanding gene regulation in computational terms (Istrail et al., 2007) or calculating the computing power intrinsic to the 3-D form of a neuron’s dendritic tree (Moldwin and Segev, 2020; Beniaguev et al., 2021). But today the existence of these distinct cellular computational mechanisms is uncontroversial, and their operational principles are now largely understood by insiders in the respective fields – molecular cell biology, developmental biology, and neuronal biophysics, respectively (Koch, 1997; Levine and Davidson, 2005; Davidson and Erwin, 2006; Bray, 2009; Cuntz et al., 2014a).

My key point in this paper is that, when we put these pieces together, the picture of neural computation that emerges is one that differs radically from both standard artificial neural networks, and more broadly from contemporary silicon-based computer technology. Since contemporary cognitive neuroscience relies heavily on both ANNs and computer metaphors (e.g., “hardware vs. software”), this has important implications for major issues in the cognitive sciences, including issues concerning digital (“symbolic”) vs. analog computation (cf. Dehaene et al., 2022). Furthermore, because single-cell computation is where biochemical/genetic and electrochemical/synaptic information processing intersect, the more inclusive conception of neuronal computation I will advance here has crucial implications for our understanding of cognition and memory, and their evolution in our own and other species.

### A brief history of synaptocentrism: Hebbian synapses and point neurons

Cajal placed strong emphasis on the complex shape of neurons, and spent much of his life documenting the rich and diverse tree-like structures of neurons throughout the brain (Ramón y Cajal, 1894–2004). Nonetheless, neural network modelers have generally treated neurons as highly simplified “units” since the foundation of the discipline by McCulloch and Pitts (1943), who modeled neurons as simple devices that sum their weighted inputs and apply a nonlinear threshold to this weighted sum to compute their binary output (McCulloch and Pitts, 1943) – in mathematical terms, they simply compute a thresholded dot-product. The focus of McCulloch & Pitts was on the computations performed by the network, not any single neuron.

This conception gained additional support with the supposition of Donald Hebb that a synaptic connection between two neurons will be strengthened when those two neurons fire simultaneously (Hebb, 1949). This now-famous “Hebbian learning rule” has become a centerpiece of theoretical neuroscience, and again focused attention away from cells or their structure and onto the connective network between cells. The discovery by neuroscientists of long-term potentiation (LTP) or depression (LTD) in the 1970s (Bliss and Lømo, 1973) offered empirical support for Hebb’s supposition, and seemed to cement a belief in an all-powerful role of the synapse in learning.

Once computer-modeling of neural networks became feasible, this synaptocentric model, and a connectionist focus on networks rather than cells continued (Bishop, 1995; Segev, 1998). More explicitly, the sole function of the “units” making up both early (e.g., perceptron) and contemporary (e.g., deep neural network) models is essentially “integrate-and-fire”: each unit performs a linear summation of the products of its input and the corresponding synaptic weights, subjects this sum to a nonlinear (often sigmoidal) threshold function, and outputs a binary spike when this weighted sum exceeds some threshold value (Rosenblatt, 1957; Rummelhart and McClelland, 1986).

The exciting advance allowed by early perceptron models was to allow “learning” via synaptic plasticity: the synaptic weights could be adjusted algorithmically to allow the model to “learn” some set of inputs as positive, and fire preferentially to those stimuli. These early network models were useful classifiers, could be applied to arbitrary digital inputs, and intriguingly showed some similarity to humans in their classification performance. Nonetheless, for many reasons (*cf.* Minsky and Papert, 1969) these earliest two-layer models did not take off.

The next wave of neural modeling built on a new and highly effective algorithm (back-propagation of error) to adjust synaptic weights throughout a complex, multi-layer network. This integration of Hebbian associative memory via synaptic plasticity with the integrate- and-fire “neuron” generated what I will call the “standard model” for artificial neural networks (ANNs) today, first in the guise of three-layer networks (with one hidden layer), and now as “deep neural networks” of many flavors that have scores or even hundreds of layers. Despite a superficial variety, the underlying computational units or “neurons” in these deep neural networks have changed little in the eighty years since McCulloch and Pitts (1943). Computational neuroscientists refer to these fictitious integrate-and-fire units as “point neurons,” to emphasize their difference from the complex and beautiful cells that actually make up our brains.

It was already clear by the 1980s that the biophysics of neurons supported a much richer set of computational primitives than a point neuron (Poggio and Torre, 1978; Torre and Poggio, 1978), and by the 1990s comprehensive lists of these possibilities were already available (Churchland and Sejnowski, 1992; Koch, 1999). In addition to Hebbian memory via synaptic plasticity, these known biophysical computation mechanisms include coincidence detection at synaptic or whole cell levels (important for temporal coding), AND-NOT logic via shunting inhibition in dendritic trees, multiplication by active (voltage-dependent) dendritic currents, and Ca++ − mediated firing propensity of the cell (“excitability”). Despite having powerful and attractive computational properties (reviewed in Mel, 1994; Koch, 1999), and being explored in hundreds of simulations and in widely used modeling frameworks (cf., Tikidji-Hamburyan et al., 2017), these additional computational mechanisms have never made it into the standard model point neuron ubiquitous in contemporary ANNs and beloved of cognitive scientists. All learning in such networks occurs by modifying synaptic weights, and neglects alternative forms of plasticity: they are “synaptocentric” models of learning and memory.

Despite this commonality, today’s impressive deep learning models have moved in a direction of increasing biological plausibility. Typically in early connectionist models, the unit-to-unit connectivity pattern was either random or complete (full connectivity), differing sharply from the underlying biology of brains, where most connections are local, and most neurons remain completely unconnected (Levy and Reyes, 2012; Markov et al., 2014). Modern deep networks (e.g., convolutional neural networks) are more biological, in that each unit in such a network has only a local connectivity pattern with those in adjacent networks. This enables both rapid computation (e.g., via matrix multiplication using GPUs) and a much greater number of layers in such networks (hence the term “deep”). Nonetheless, these models retain point neurons and a reliance on a biologically unrealistic back-propagation of error (cf. Lillicrap et al., 2020). Although it is clear that the brain transmits error signals between neurons in different regions across the global scale (Wolpert et al., 1995; Clark, 2013; Roth et al., 2016; Friston, 2018), there are no biologically plausible models by which synapse-specific error signals could be propagated across multiple neurons (Lillicrap et al., 2020). Intriguingly, Senn and colleagues have shown that, in models that move beyond point neurons to active dendritic computation (see below), back-propagation of error within dendrites is both biologically plausible and computationally powerful (Schiess et al., 2018; Wybo et al., 2023), a point further explored below.

For many engineers, the question of how closely their networks model biological neurons is irrelevant: practical machine learning with point neurons works well enough for many purposes. Perhaps more surprising, many neuroscientists have also come to accept the synaptocentric perspective, in which synaptic plasticity is the sole (or at least main) mechanism underlying learning and memory, despite long-known neuroscientific evidence suggesting a much richer model of neuronal computation. This may be partially due to the discovery of NMDA receptor-dependent LTP, which offered an exciting molecular mechanism by which the Hebbian dictum that “neurons that fire together wire together” could be implemented in actual synapses. But even NMDA-dependent LTP, we now know, involves a host of specific underlying mechanisms and different biophysical substrates (Malenka and Bear, 2004): there are many ways to update synaptic strengths, each having differing properties. Furthermore, it is equally clear today that synapses themselves are nonlinear, so the traditional linear dot-product of synaptic inputs and synaptic weights omits specific and important forms of neuronal computation observed in real brains (Zador, 2000). Given this weight of neurobiological evidence, it seems imperative to ask what is being omitted in current synaptocentric models of memory (information storage), and/or what is missing in models of computation based on point neurons.

### Beyond synaptocentrism

In addition to the “bottom-up” neurobiological evidence discussed above, equally pressing reasons to re-evaluate synaptocentrism have come from cognitive science, “top-down.” This re-evaluation has been recently spurred by a series of critiques led by the comparative psychologist Randy Gallistel (Gallistel and King, 2010; Gallistel, 2017, 2020; Langille and Gallistel, 2020). This recent backlash against synaptocentrism builds on a much older long-running debate between associationism and other models of cognition (symbolic, computational and cognitive models), itself hearkening back to earlier debates between behaviorists and early cognitive scientists concerning the very existence of cognitive elements such as goals, plans, emotions or memories (Gardner, 1985). Briefly, Gallistel’s critique is that what is stored in memory, in humans or in animals, are not associations between events, but concepts and facts about the world (distances, amounts, identities, words, locations, etc.). It is argued that such facts, and in particular numerical values, cannot be captured solely by associations, and that associationism is thus a deeply inadequate model of memory or of cognition (*cf.* Bever et al., 1968; Fodor and Pylyshyn, 1988; Fodor and McLaughlin, 1990). To the extent that this critique is valid, *any* model of memory that relies entirely on Hebbian associations will fail, and some form of symbolic computation is required to understand cognition in any species (cf. Trettenbrein, 2016; Prasada, 2021).

The practicing neuroscientist or psychologist may be tempted to dismiss such long-running arguments as philosophical hair-splitting (indeed, many of the protagonists in the traditional debate were philosophers), or to consider it a matter of taste whether one favors associationist or symbolic models of the mind. Indeed, the answer may come down to a question of level of analysis: associations at an implementational level may encode symbols at a higher computational level (Smolensky, 1988; Chalmers, 1993). However, even as simplified models of the brain, there are a host of other problems with a synaptocentric view, strongly grounded in neurobiology, that add bottom-up fuel to this cognitively oriented debate (Arshavsky, 2006; Kastellakis et al., 2015; Trettenbrein, 2016; Langille and Gallistel, 2020; Poirazi and Papoutsi, 2020; Gershman, 2023).

One of the key problems with synaptic plasticity as the locus of memory is, ironically, that synapses are *too* plastic [cogently summarized by Gershman (2023)], while memories can last a lifetime. For example, long-term memories, whether implicit memories such as a major early life event experience, or implicit knowledge such as motor skills, or word meanings learned at a few years of age, can persist for an individual’s entire lifespan (Poeppel and Idsardi, 2022). From this viewpoint, “long-term” potentiation is a misnomer, since the effects of glutamatergic LTP at the synapse last hours or at most days (Malenka and Bear, 2004). In fact, synaptic weights are constantly changing [e.g., due to spike-timing dependent plasticity (Bi and Poo, 2001)], and the dendritic spines that house most excitatory synapses are in a constant state of flux (Loewenstein et al., 2015). Changes in dendritic spine morphology directly reflect learning and memory [e.g., Roberts et al., 2010; Ashokan et al., 2018], but even the longest-lasting changes in dendritic spine morphology probably last at most a few months (Yang et al., 2009). Problematically, synapses and synaptic spines require a constant and relatively high metabolic cost to maintain their current state, relative to some other loci of memory discussed below. This plasticity and variability on a short time scale, combined with their high metabolic cost and various other “sins” (cf. Arshavsky, 2006), conspire to suggest that synapses are poorly suited to represent the sole and final locus of long-term memory over weeks or years (cf. Gallistel, 2020; Poeppel and Idsardi, 2022).

This growing weight of evidence has led most of the authors cited above to argue that synapses cannot form the sole basis of memory. Although none of these critics deny the fact of synaptic plasticity via LTP/LTD, nor deny that it plays a role in memory and learning, all of these lines of argument suggest that other, more stable and low-cost, biophysical mechanisms must also be involved in long-term memory. Indeed, these considerations have led some authors to suggest that long-term memory must somehow be stored intracellularly, in the form of RNA or DNA based codes (Gallistel and King, 2010; Gallistel, 2020). At first blush, this is an appealing idea, because nucleic acids represent the ultimate low-cost, long-lasting biological mechanism for information storage. Unfortunately, there is no known mechanism by which information stored temporarily in patterns of synaptic weights could be “translated” into base-pair encodings, and the very idea of such a back-translated encoding goes against most of what is currently known about the molecular biology of the cell. These and other facts have led some commentators to entirely reject Gallistel’s argument, I think prematurely (e.g., Dayan, 2009). But I will argue that acknowledging the weaknesses of the Hebbian synapse and synaptocentric arguments does not require embracing any hypothetical undiscovered reverse-transcription based memory mechanisms.

My goal in the rest of this paper is to show how current knowledge of neuronal biology allows us to move beyond synaptocentric conceptions of memory and point neurons, and to address and answer Gallistel’s challenge based on established biological facts and computational concepts. I will first show how a biophysically grounded model of cellular computation in real brains, richer than that envisioned by standard point neurons, combined with contemporary understanding of genomic computation, provides fresh answers to both the storage and computation questions. I will end by considering the cognitive and evolutionary implications of such a more biologically realistic, cell-based computational viewpoint.

## Cellular computation: a computer in every cell

The central point of the next sections is that each individual neuron is a powerful computer in its own right, with a computational power roughly equivalent to an entire ANN (Poirazi et al., 2003; Moldwin and Segev, 2020; Beniaguev et al., 2021), and an information storage capacity much greater than the 1–10 kB stored in a neuron’s 1,000–10,000 synaptic weights (Poirazi and Mel, 2001; Bray, 2009; Brenner, 2012; Fitch, 2021). While many of these ideas were first advanced by modelers, there are now many empirical studies confirming these early suppositions in actual neural systems (ably reviewed in Kastellakis et al., 2023). The existence of dendritic computational phenomena, including dendritic spikes and active conductances at dendritic branches (Gidon et al., 2020), means that many Hebbian phenomena previously thought to require metabolically expensive whole-cell firing, such as LTP and LTD, can in fact occur at a local, dendritic level. Conceptually, this is equivalent to adding a second layer of computation to the traditional Hebbian/connectionist model, intervening between the synapses and the whole cell. This new conception renders biological neuronal networks much more energetically efficient than previously though (a key evolutionary desideratum). Furthermore, because neighboring synapses act cooperatively, spatial localization of connections can now play a central role in cellular computation, such that inputs that are contextually or conceptually related cluster together in space on the dendritic tree (Kastellakis et al., 2023).

From this updated biological perspective, trying to understand brain function without attending to dendritic structure is like trying to understand a community based on a listing of its individual members, without attending to their personalities, where they live, or their family and neighborhood dynamics.

There are multiple distinct biophysical systems underlying this cellular computational power and storage capacity, each with its own properties. These include electrodynamic processes, short-term biochemical computation or “wetware,” and longer-term gene expression systems. By “electrodynamic processes” I mean the neuronal biophysics traditionally studied by cellular neurophysiologists – ion currents, membrane potentials, and voltage- or ligand-gated ion channels – but crucially incorporating the computational role of the complex 3-D branching structure of the cell, which has powerful effects on its input–output relations (Koch et al., 1982; Koch and Segev, 2000; Moldwin and Segev, 2020; Kastellakis et al., 2023). In the short-term (“fast”) biochemical category, I include all of those cell-internal processes encompassed by Dennis Bray’s term “wetware” – protein marking via phosphorylation, methylation and other processes – and active protein trafficking via the cytoskeleton. Wetware makes contact with electrodynamics in that internal ion flows, especially Ca++ flows, often play an important cell-internal signaling role (Malenka and Bear, 2004) that has lasting effects on future electrodynamic responses. Finally, gene expression involves slower biochemical computation (*cf.* Istrail et al., 2007; Brenner, 2012), involving genomic marking (via methylation or chromatin modifications) and is most clearly reflected in the cell’s current transcriptome (the dynamic set of RNA transcripts in the cell).

Each of these systems mutually influences the others, making the borders between them somewhat fuzzy. Electrodynamic phenomena affect internal biochemistry, and both affect gene expression, which in turn has powerful reciprocal influences on electrodynamics and “wetware.” I nonetheless distinguish them for several reasons. First, although there is information storage (“memory”) at each of these levels, the mechanisms used are quite different: chromatin modification, protein phosphorylation, and dendritic or synaptic morphology are conceptually and biophysically distinct, and operate on rather different time scales. Second, from an empirical viewpoint, the data used to study cell function at each level differs: we use multi-electrodes or calcium imaging to measure electrodynamics, but use single-cell transcriptomics to understand gene expression patterns. This makes collating these empirical data a challenge in itself, but one which can now be met using existing methodologies (cf. Nandi et al., 2022). Finally, these distinct mechanisms operate on different (but overlapping) characteristic time scales, with electrodynamics being fastest, gene expression slowest, and wetware somewhere in between. This implies that we may usefully analyze functionality at fast levels by modeling the slower level(s) as fixed state variables characterizing that neuron. For example, in analyzing electrodynamics, we can adopt a millisecond timescale and can treat the cell’s current form, wetware state, and transcriptome as unchanging.

It is important to recognize that both wetware and gene expression patterns are properties of all cells, not just neurons (Bray, 2009; Brenner, 2012), but are nonetheless centrally relevant to neurons, and thus to neuroscience. Wetware is a form of non-synaptic computation particularly important in understanding behavior in single-celled organisms such as bacteria or *Paramecium*, which obviously lack both neurons and synapses, but are still capable of complex-goal directed behaviors and, in the case of single-celled eukaryotes, learning and memory (“single-cell cognition,” cf. Tang and Marshall, 2018; Marshall, 2019; Dussutour, 2021; Gershman et al., 2021). Because I have recently reviewed these data, and their implications for evolutionary neuroscience, elsewhere (Fitch, 2021), I will simply note here that such data in themselves call any strictly synaptocentric model of memory and computation into question (cf. Gershman et al., 2021).

I will now briefly survey the key properties of each of these four distinct computational mechanisms. I will begin with the best-understood and least controversial level – gene expression – and end with 3D electrodynamics, whose detailed computational properties are less clear, and are a topic of current active research.

### Genomic computation

Virtually all of the cells in our body share an identical copy of our genome (red blood cells are an exception). The distinctions between different cell types are a result of variation in gene expression between cells. Metaphorically speaking, all cells possess the same library, but each cell type reads a different subset of the books within. Which books are read (which genes are expressed) is determined by the regulatory genome, and the cell’s current regulatory state. The regulatory genome includes non-coding DNA binding sites in the neighborhood of protein-coding genes, whose bound or unbound state controls the expression of neighboring protein-coding genes.

The key computational elements for genomic computation are several hundred thousand *cis*-regulatory modules, including sections of DNA to which transcription factors can bind, thus enhancing or suppressing expression of the neighboring genes on the same strand of DNA (Britten and Davidson, 1969; Davidson, 2006; Istrail et al., 2007). Transcription factors are short proteins that selectively bind to DNA at specific binding motifs within a *cis*-regulatory module, controlling the rate of transcription of genes in their vicinity. There are roughly 1,600 different transcription factors in humans (Lambert et al., 2018). For brevity, I will term an entire set of regulatory sites (containing transcription-factor binding sites and protein coding genes) a “gene expression module” or GEM (roughly equivalent to an “operon” in bacterial genetics).

Each of the 10^5^ GEMs in our genome contains multiple control regions involving multiple transcription factors, which can interact in complex ways. In particular, activation of a GEM typically requires binding of multiple factors, that can work additively or oppose each other. This means that the entire gene regulatory system can be analyzed in computational terms (Istrail et al., 2007; Brenner, 2012) as a set of interacting AND, OR, and NOT gates (along with more complex logical combinations). We can thus picture the current levels of transcription factors as “input,” the current state of binding as “memory,” and the resulting gene expression (the current transcriptome) as “output.” However, we cannot draw a clear hardware/software distinction for this form of computing: memory and computation for one GEM are co-localized to small regions of DNA, and GEMS are spread throughout the genome. The end result of this computational process will be a set of RNA transcripts that are transported out of the nucleus, where (after further editing) they will be translated into proteins. Roughly 10% of this RNA codes for the transcription factors, which can then bind to DNA throughout the genome. The remaining RNA codes for the molecular machinery for other cell functions (including neurotransmitters and neurotransmitter receptors, and the synthesis machinery for other mechanisms that will control neuronal electrodynamics).

Binding of transcription factors to DNA is relatively stable over periods of hours or days, but remains stochastic and unlikely to provide a truly long-term memory over years. A second related source of information storage is provided by chromatin modifications (such as DNA methylation, or histone acetylation: Watson et al., 2014), today often termed “epigenetic” changes (Holliday, 2006). Most of the DNA in a cell is tightly wound around protein complexes termed histones, and in a differentiated cell only a small subset of the DNA is normally unpacked and exposed for binding or transcription. Continuing the library metaphor, the DNA library involves a rolling shelf system, where most of the shelves in the library are pushed against one another and unavailable for browsing; chromatin modification processes can open some of these sections up.

Chromatin modifications provide a form of cell-internal storage that plays a central role in development: the identity of a cell (as liver, muscle or any of several thousand types of neuron) is essentially “coded” by what portions of its genome are “open” for binding and transcription. Once a cell differentiates, chromatin modifications can remain in place for the life of the cell – which for a neuron is measured in decades. This form of memory is thus extremely long-lasting and, once established, can be maintained at virtually no metabolic cost. These properties make chromatin methylation or acetylation an ideal mechanism for long-term memory storage at the cellular level. Indeed, methylation patterns can be transferred from the mother’s egg to her offspring, extending beyond the lifespan of a single organism (hence the term “epigenetic”). However, it remains unclear whether epigenetic changes at the whole cell level could influence specific connection strengths (Campbell and Wood, 2019).

The memory capacity of the entire gene regulatory/epigenetic system is vast in principle. There are roughly 10^5^ cis-regulatory modules, and if each could be bound or unbound independently as a binary variable, it would yield 10^5^ bits, meaning 2^10000^ or ~ 10^3000^ possible states! Similarly, each chromosome contains hundreds of thousands of histones, and again each can be in an open or closed state. Despite this vast potential, constraints on the epigenetic system, particularly the limited number of transcription factors, severely constrain this possible state space. Nonetheless, even basing a conservative lower bound on the number of transcription factors (1600), each treated as a binary variable (expressed or not) yields 2^1600^ or ~ 10^51^ possibilities – vastly more than the number of synapses (roughly 10^14^ in the human brain). Of course, the state of these GEMs is crucial to all aspects of cellular function and developmental biology (Howard and Davidson, 2004), and cannot be simply used as a memory storage device for arbitrary cell-specific information. Furthermore, we know that epigenetic factors and gene expression patterns play a key role in determining the *type* of neuron during early development, but it remains unclear to what extent these systems encode the specific past history of individual cells during adulthood. But if even a tiny fraction of these DNA binding sites or histones were available to store information about a particular neuron’s past state (and thus the organism’s past experience), it would provide a formidable auxiliary memory that is digital, cheap to modify, and highly stable over time.

Returning to issues of computation, there is a long tradition of seeing gene regulation in computational terms, dating back to the discovery of the first molecular “switch” in bacteria, the *lac*-operon (Jacob and Monod, 1961). But this computational perspective was still unfamiliar enough in 2012 for its importance to be stressed by the Nobel-prize winning molecular biologist Sydney Brenner (Brenner, 2012). Computational perspectives on gene regulation play a central role in the burgeoning field of synthetic biology (Benenson, 2012). Perhaps the clearest codification of gene regulation in computational terms is due to Eric Davidson and colleagues (Istrail et al., 2007), who stressed the deep conceptual similarities between genomic computation and the more familiar electrical computation in silicon, but also discussed some important ways in which they differ. Among these differences, in artificial computers information is transmitted point-to-point by wires, but in cells the means of information transmission is diffusion of small molecules. Diffusion can be quite rapid in the case of local communication within the nucleus, or within a small bacterial cell (cf. Bray, 1995, 2009), or quite slow along the many millimeters of some axons. The cell’s system is also massively parallel: many molecules diffuse to many different DNA binding sites simultaneously (Istrail et al., 2007). Finally, genomic computation is highly redundant: there are many routes to achieve the same transcriptional outcome, which makes the system highly robust to disturbances, and thus stable across a wide range of circumstances. These are all in sharp contrast to contemporary von Neumann computer architectures, which separate hardware from software, communicate with point-to-point specificity along wires, utilize a single uniform communication currency (current or voltage), and typically operate serially.

Despite these differences, the gene regulatory system is an example of “natural computation” involving both information storage (memory) and processing (computation), best understood in computational terms (Istrail et al., 2007; Brenner, 2012). Although this computational system characterizes *any* eukaryotic cell (from yeast to liver cells and including neurons), the key implication for the topic of this paper is that genomic computation provides an increasingly well-understood computational system that equips individual neurons with powerful computational resources, including several forms of long-lasting memory that are independent from, and much more stable than, synaptic forms of memory. They are however strongly influenced by (and therefore coupled to) the cell’s electrodynamic history and current biochemical state, to which we now turn.

### Wetware: rapid biochemical computation

A second biochemical computation system characterizing living cells is encompassed by cell biologist Dennis Bray’s term “wetware”: the set of signaling proteins which are specialized to store, transfer and process information within a cell (Bray, 2009). These signaling proteins are able store information (for example via protein phosphorylation or methylation) and transmit it (via diffusion of cell-internal signaling molecules termed “second messengers,” for example cyclic AMP), and are arranged into biochemical ‘circuits’ that can compute various types of simple functions (e.g., amplification, addition and multiplication) (Bray, 1995; Benenson, 2012). Several systems of wetware are quite well-understood, such as the mechanism underlying bacterial chemotaxis (cf. Bray, 2009). In single-celled eukaryotes (e.g., *Paramecium* or *Stentor*), wetware and gene expression are the only computational systems available, and enable these organisms to sense their environment, store information, and control action. These two systems thus represent the core mechanisms underlying the impressive feats of learning and memory documented in single-celled eukaryotes (Tang and Marshall, 2018; Dexter et al., 2019; Marshall, 2019; Dussutour, 2021; Fitch, 2021; Gershman et al., 2021). Crucially, neurons inherit this computational machinery simply by virtue of being eukaryotic cells. Many of these wetware systems (e.g., G-proteins and cyclic nucleotides such as cAMP and cGMP) play a ubiquitous and well-studied role in neurophysiology (Schulman, 2004), and are so familiar to cellular neurophysiologists that they are seen simply as necessary background knowledge from molecular biology. Despite this importance, these are rarely considered in computational terms, and their potential roles in neuronal computation thus often remain either unmentioned (Gazzaniga et al., 1998) or implicit (Bear et al., 2001) in neuroscience textbooks.

In neurons, the cell-signaling pathways that together comprise wetware play a critical role as the bridge between fleeting electrodynamic phenomena such as EPSPs, ion influx, or action potentials, and longer-term changes in gene expression. This is best understood in the context of LTP, illustrating how electrodynamic phenomena (e.g., correlated Hebbian firing) are translated into longer-lasting changes in synaptic and dendritic morphology. In glutamatergic LTP, the NMDA receptor serves as a molecular AND gate that only opens when a glutamate molecule is bound to it and an action potential fired by the host cell. When this occurs, it allows calcium ions to flow into the cell which provides a trigger for calcium-dependent protein kinases (e.g., CaMKII) that play a role in synaptic weight modification in LTP (Malenka and Bear, 2004; Kastellakis et al., 2023). Such NMDA-mediated changes were once thought to be restricted to a single synapse, but it is now clear that they also strongly influence neighboring synapses on the same dendrite (Kastellakis et al., 2023; Wybo et al., 2023) - so-called “heterosynaptic plasticity.” Fortunately then, all of the virtues of this well-known plasticity mechanism extend nicely beyond the synaptocentric perspective to include the dendrite-focused viewpoint I argue for here.

A host of other molecular mechanisms that bridge between rapid electrodynamic events and long-lasting changes in cell form via intracellular wetware are currently the topic of intense study. For example, the last decades have made clear that the process of translating genes into protein from messenger RNAs is distributed thoughout the dendritic arbor (rather than limited to the cell body, as previously thought) (Sutton and Schuman, 2006). This local translation supports an activity-dependent protein synthesis, which in some cases (e.g., the *Arc* gene) can lead to further transcription of the gene. Such dendritically localized processes are now thought to be crucial to explaining how short term changes in electrodynamics can lead to the long-term stability needed for lasting memories to form (*cf.* Das et al., 2023).

For our purposes here, the crucial point concerning neuronal wetware is that it provides a powerful cell-internal computational mechanism that both responds to neuronal electrodynamics, and causally affect gene expression and cell morphology, using physical mechanisms independent from either of them. It operates on timescales intermediate between these two, and thus provides an important conceptual and informational bridge between these other two computational levels.

### The connectome: dynamic neuronal connectivity and the brain’s “wiring diagram”

The above discussion of genomic computation (e.g., Istrail et al., 2007) focused on its role within individual cells, but genomic computation plays an equally important role in creating the whole-brain wiring diagram in the first place. Despite the power of individual neurons (see below), they never work alone but rather function in complex networks. The fact that neurons are dynamic agents, extending axons and forming connections with other cells during development, was part and parcel of Cajal’s introduction of the neuron doctrine (Finger, 2000), and the significance of this morphological plasticity for brain wiring has long been recognized. For many years, clear evidence for this was limited to the developing nervous system or recovery from trauma. Unambiguous evidence that axonal and dendritic plasticity also play a key role in adult learning and memory has only recently become available (e.g., Biane et al., 2019; Kastellakis et al., 2023). This suggests that the details of connections between neurons - overall neural architecture - potentially play a central role not just in neural development, but in ongoing neural computation, information storage, plasticity, and learning in the adult brain (Van Kerkoerle et al., 2018).

Despite my focus in this paper on the computational power of single cells, neurons in brains are of course members of large networks of interconnected cells, and neural computation in its fullness must be understood in ensemble terms of network computation (Libedinsky, 2023). It is thus a misconception to focus only on one or the other of these two levels (contra Barack and Krakauer, 2021). A crucial factor in understanding these network-level computations is the specific point-to-point connectivity between neurons, how it develops, and how it changes based on experience.

The brain’s wiring diagram – the set of connections between neurons – is now widely referred to as the “connectome.” Despite the new name, understanding this wiring diagram has been at the heart of neuroscience since its inception. Throughout most of the history of this field, mapping connections required tract tracing – a laborious and time-consuming process that typically involved brain injections in living animals and later sacrificing them for histology (Markov et al., 2014). Today, a host of new tools makes studying the connectome much easier. The primary tools include single-cell transcriptomics, which allow us to study the gene expression patterns that control brain wiring, genetic engineering to study the global effects of single-gene knockouts or enhancements, and/or morphology and connectivity of selected neuron classes. While less accurate, whole brain MRI scanning and analysis of large fiber tracts using diffusion tensor imaging provide us with a global map of connectivity of the entire brain, and can be used in living subjects including humans (Rilling et al., 2008; Makuuchi et al., 2009; Jbabdi and Johansen-Berg, 2011). These new tools provide powerful, multi-scale analysis of connectomes in different species, different individuals within a species, and even developmental time courses of the same individual across development. Connectomics has come of age.

It is now increasingly recognized that the connectome is dynamic at multiple spatial and temporal scales, and that this plasticity plays a role both during development and in adult learning (Takeichi, 2007; Hirano and Takeichi, 2012). Starting with brain development, expression of cell-adhesion molecules such as cadherins plays a central and ubiquitous role in neurogenesis, migration patterns of neuronal precursor cells, and formation of high-specificity axonal connections, as well as key roles in the complex tree structure of individual cells (e.g., axonal and dendritic tree complexity) (Hirano and Takeichi, 2012). The cadherins are a large family of molecules that play crucial roles in development by regulating cell differentiation, cell migration, and cell-to-cell contact including synapses. Their roles during development are complex and diverse (Hirano and Takeichi, 2012), and so I only summarize a few highlights here.

In building the brain’s “wiring diagram” – the connectome *per se* – cadherins and similar molecules play important roles in guiding axonal growth trajectories and in synapse formation during development. These appear to be mediated by a so-called “adhesion code” (Krishna-K et al., 2011; Hirano and Takeichi, 2012) determined by different combinations of cadherins (and other cell-surface molecules). During brain wiring, axonal growth cones extend out from young neurons and guide axonal growth by sensing environmental guidance cues – by “sniffing” their way through the brain (Tessier-Lavigne and Goodman, 1996). A host of different signaling molecules, including cadherins, semaphorins, and others, play key roles in this process (Redies et al., 2003; Tran et al., 2007), and depending on the match between the axon and its potential targets, may repel the growth cone, or attract it. Once the growth cone arrives at a potential synaptic target, the match or mismatch of cell-surface proteins can further determine whether or not synapses are formed. Because there are more than 100 cadherin types in vertebrates, and they can be co-expressed in arbitrary patterns in different cells, this provides a rich combinatorial code that can determine cell-to-cell and region-to-region connectivity with high precision (cf. Bekirov et al., 2008). These signaling molecules also have far-reaching effects within the cell, particularly differentiation into neuronal subtypes and/or stabilization or dissolution of the cell’s internal actin cytoskeleton. That is, once a cell has formed the correct connections, it “senses” this fact, and can then differentiate into its final terminal cell type, expressing the correct neurotransmitters and receptors, and stabilizing its form (potentially by retracting other, dis-preferred connections).

An excellent example of the role of dynamic cadherin expression in the establishment of the fundamental connectivity in the brain comes from the song-learning system in songbirds, where changes in the timing and location of cadherin expression play a central role in creating the song-system wiring diagram (Matsunaga et al., 2006; Matsunaga and Okanoya, 2008, 2009). To properly learn their songs, young songbirds require early exposure to their species-typical song, and young birds store these songs as templates before they begin singing themselves. When older, the bird then enters the sensorimotor or “babbling” stage, where it begins producing song itself and converging, over weeks, to a final song that matches the learned template(s). This requires synaptic connections between sensory, cognitive and motor regions. When the maturing bird begins to practice singing, cells in a key song motor nucleus switch from expressing a repellent cadherin-7 to a “matching” cadherin-6, creating a hand-shake signal which induces synapses with axons projecting from higher-order song regions to form. This is an excellent example where the gene expression (genomic computation) has a direct causal effect on connectivity, and where both map nicely onto whole-organism behavior. This is just one of many well-studied examples showing that cadherin expression patterns play key roles in long-range connectivity and synapse formation in the developing brain (Takeichi, 2007; Matsunaga and Okanoya, 2008), and thus in the creation of basic brain circuitry (cf. Hirano and Takeichi, 2012).

Similar mechanisms also play a role in determining the detailed form of individual neurons, particularly the structure and complexity (e.g., branching patterns) of the dendritic and axonal trees. For example, N-cadherin plays an important role in determining retinal receptive field sizes, by controlling attachment between retinal horizontal cells to photoreceptors (Tanabe et al., 2006), and a combined code involving co-expression of N-cadherin and cadherin-8 plays a key role in connectivity and arborization in the hippocampal mossy fiber pathway (Bekirov et al., 2008). Cadherins also play important roles in stabilizing synapses once they have formed (Brigidi and Bamji, 2011).

Summarizing the developmental data discussed so far, the expression of different cell adhesion molecules in specific cells plays a key role in laying out the initial wiring of the brain, both via early cell migration and in later growth-cone based guidance of axonal connectivity and synapse formation. The same or closely related factors also play a role in generating the dendritic and axonal tree form, which both play key roles in determining the computational role of single cells within this network (as detailed in the next section, cf. Shepherd, 2004). These are all ultimately controlled by gene expression patterns at the single-cell level. Thus, the underlying gene expression patterns that generate the connectome blur the line between cell-internal and cell-external computational mechanisms.

Regarding the role of such connectomic changes in adulthood, their potential funtion in adult long-term learning and memory remains less well-understood. The formation of new synapses at new dendritic spines is well-documented (Hickmott and Ethell, 2006). Data from birds during song learning demonstrates the computational role of dendritic spine plasticity in fully grown young birds (Roberts et al., 2010). Strong recent evidence comes from a study by Biane and colleagues which demonstrated that motor cortical connectivity is modified during motor learning, that these modifications are restricted to the relevant microcircuits, and that blocking neuronal plasticity impairs learning (Biane et al., 2019). Furthermore, such dynamic changes in connectivity have also been shown for axonal arbors in cortex, where both sprouting and pruning of new axonal branches are seen during perceptual learning in macaque visual cortex (Van Kerkoerle et al., 2018). These and other data indicate that the connectome remains dynamic throughout life, and thus that specific cell-to-cell connectivity continues to play an important role in neural computation and plasticity during adulthood.

From a computational perspective, the information storage capacity of the connectome is vast. Although wiring still involves synapses, the connectome involves the absolute presence or absence of synapses, rather than changes in synaptic weights of existing synapses (Van Kerkoerle et al., 2018). The connectivity matrix between cells provides an additional medium for memory storage (Fitch, 2021), which has a discrete binary character rather than the continuous values of synaptic weights. While this connectivity remains “synaptocentric” in one sense, such all-or-nothing connections, once formed, can be inexpensively maintained via thermodynamically stable cell-adhesion molecules such as cadherins (Takeichi, 2007; Hirano and Takeichi, 2012). This storage medium is both metabolically cheap and stable over months, and can be maintained by cell-internal factors including gene expression patterns yielding matching adhesion molecules in the two connected cells (*cf.* Matsunaga et al., 2006; Matsunaga and Okanoya, 2008) and/or stabilization of the internal cytoskeleton.

Graph-theoretically, this form of information storage could be captured by a vast neuron-to-neuron connectivity matrix where most of the connections are set to zero (no connection) – an extremely sparse matrix (Levy and Reyes, 2012). While applicable to very small nervous systems (like that of *C. elegans*, with 302 neurons, Varshney et al., 2011), applying this brute-force approach to human cortex would require an impractical 10^10^ × 10^10^ connectivity matrix. Although absurd from an implementational viewpoint it offers a first suggestion of the information capacity of the cortical connectome: 10^20^ bits! Of course, to a large extent the developmental program that constrains connectivity among different brain regions is fixed within a species by evolution, so many of these theoretical connections are probably unreachable in practice (Markov et al., 2014). However, even a tiny fraction of these possible connections would provide a formidable memory store if they remain settable in adulthood. If each of 10^10^ cortical neurons retained a dynamic capacity to form or retract synapses on each of 10 recipients, this would still provide 10^11^ bits of metabolically cheap, thermodynamically stable, long-term information storage. Thus, considering the “wiring diagram” of the brain to be fixed would yield a massive underestimate of the potential capacity of neural storage at the cell-to-cell level.

### Electrodynamics: dendritic computation and deep neurons

**Figure 1:**
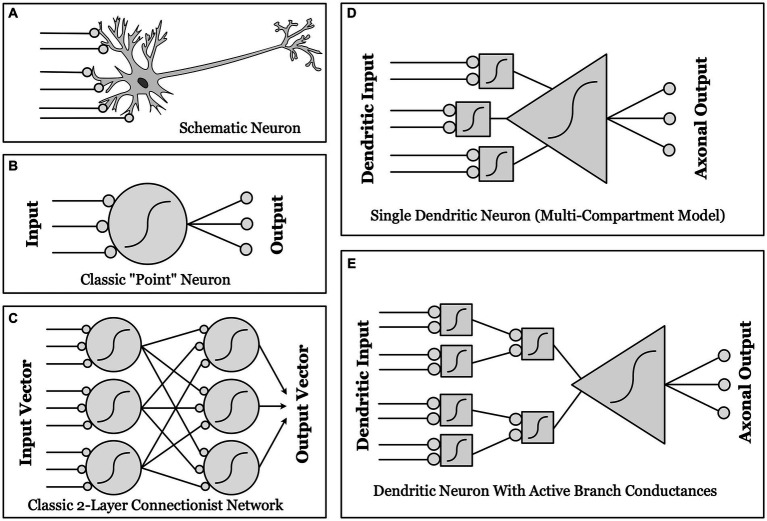
Schematic illustrations of different conceptualizations of neural computation. **(A)** Real biological neurons receive synapses (lines ending in gray circles) onto complex, branched dendrites, which join at a cell body, which then projects one or more axons to synapse upon other neurons. **(B)** “Point neurons”: The dominant conceptualization of a “unit” in contemporary artificial neural networks (ANNs) is a simple “point neuron” which has no structure: it simply multiplies the input from each synapse by a weight, and sums these weighted inputs. If the sum is above threshold (a nonlinear function, as indicated by the sigmoid curve), the unit “fires” an output. **(C)** A classical two-layer ANN, with fully connected input and output layers made up of “point neurons”. **(D)** “Dendritic Compartmental Model”: A more accurate but still incomplete model of a neuron, represented by multiple dendrites, each computing a weighted sum of its own synaptic inputs. **(E)** “Active Branch Conductances”: The simplest computational model capable of approximating the actual complexity of biological neurons has both separate dendritic compartments, and active conductances at the branch points where dendrites join together. These support a number of separate nonlinear threshold functions before the final whole-cell threshold, thus allowing a single neuron to approximate a two-layer ANN in complexity and computational power.

Finally, I return to cellular neurophysiology to discuss the last, and most exciting, category of cell-internal computation: electrodynamics as influenced by cell morphology, and particularly the shape of the dendritic tree (Figure 1). This class of phenomena can be termed “single-cell computation” or “dendritic computation,” and is the topic of a large and fast-growing field (Häusser and Mel, 2003; Schiess et al., 2018; Gidon et al., 2020; Larkum, 2022; Kastellakis et al., 2023). Single-cell aspects of neural function are critical to the function of neural circuits, but have been consistently ignored in “standard” point-neuron models since McCulloch & Pitts (Shepherd, 2004). Neuroscientists interested in cellular biophysics have nonetheless been studying this type of computation for many decades, often under the rubric of “dendritic computation” or “active dendrites.” The many distinct categories of computation that can be carried out in dendritic trees have been surveyed in multiple excellent reviews (Koch, 1997; London and Häusser, 2005; Cazé et al., 2013; Remme and Torben-Nielsen, 2014; Poirazi and Papoutsi, 2020; Kastellakis et al., 2023), and are reviewed at book length elsewhere (Cuntz et al., 2014a). Here, I will only provide a brief overview, focusing on aspects of neuronal form that are relevant to both of Shannon’s interests: computation and information storage.

Single-cell dendritic computation has been well documented in multiple cells types throughout the brain, including Purkinje cells in the cerebellum, medium spiny neurons in the striatum, and pyramidal cells in the hippocampus and cortex (Shepherd, 2004). Single-neuron computation is thus ubiquitous. Although there was still debate in the late 1990s about whether dendrites enhance neural computation, this is no longer controversial (e.g., Borst and Egelhaaf, 1994; Segev, 1998; Guerguiev et al., 2017; Gidon et al., 2020; Kastellakis et al., 2023). This renders it rather mysterious that this entire class of cell-based neural processing continues to be essentially ignored by “neural network” modelers or cognitive neuroscientists.

I will now provide illustrative examples of the power of dendritic computing, first discussing the computations made available by “passive” dendritic trees: those that lack voltage-gated ion channels. Even the addition of a single filamentous dendrite, modeled as a passive cable, to a point neuron adds computational power (Rall, 1964). Because of conduction delays and a steady voltage drop along the length of the dendrite, the distance of a synapse from the cell body has an important effect on how excitatory post-synaptic potentials (EPSPs) propagate, and thus the likelihood that a series of EPSPs will fire the cell. For example, a collection of synapses receiving precisely the same number of EPSPs may or may not fire the cell, depending on the precise timing of this synaptic input.

If these inputs are timed and localized such that summation occurs down the length of the dendrite, the net voltage change at the soma will be greater than if they are activated randomly, or in a non-summating pattern. This allows a simple directional sensitivity in the whole cell output, where it will only fire when its inputs “move” down the dendrite rather than up it. This type of direction-sensitivity is well documented in early vision in the vertebrate retina interneurons (Cuntz et al., 2014b). A slightly more sophisticated form of selectivity, still essentially passive, can be achieved more compactly in space via impedence gradients and nonlinear ion channels (e.g., NMDA channels: Branco, 2014). These examples show that even a cell with drastically simplified dendrites possesses a greater repertoire of potential spatio-temporal patterns to which it can tune itself, for example to implement a direction-sensitive motion detector as seen in the retina of flies or vertebrates (Cuntz et al., 2014b).

A second important type of passive dendritic filtering incorporates multiple dendrites, and allows the branching structure of the dendrites to play a separate role in determining cell firing. This results from the phenomenon of “sublinear summation”: the fact that, due to membrane biophysics, the EPSPs of closely neighboring excitatory synapses on the same dendritic branch will not be fully additive (Rall, 1964; Segev, 1998). In the simplest case of a bipolar neuron with two dendritic branches, several EPSPs co-localized to one dendritic branch will not fire the cell, while the same number distributed over both branches will. This provides a mechanism by which a single neuron can implement an AND function over its two branches, only firing when both dendrites are activated (Shepherd, 2004), or more complex time-adjusted coincidence detection, as seen in the visual and auditory periphery (Borst and Egelhaaf, 1994; Agmon-Snir et al., 1998).

Although so far we have discussed summation of EPSPs, inhibitory inputs are also ubiquitous phenomena in biological neural networks. In a point neuron, inhibition is always global, and affects all of the input EPSPs equally. In reality, inhibitory inputs can play a diverse role in dendritic computing, allowing “targeted inhibition” of a particular dendritic branch (Koch et al., 1982). Because inhibitory neurons typically impinge upon their targets in multiple locations on the dendritic tree, and multiple inhibitory neurons contact each cell, this allows for more sophisticated subsetting of the dendritic computations than would be allowed by EPSPs alone.

The phenomena above all occur even in passive dendritic trees. However, the full power of dendritic computation only becomes evident when the nonlinearities added by active (voltage-sensitive) channels are considered (Poirazi and Mel, 2001; Gidon et al., 2020), along with dendritic tree structure (Moldwin and Segev, 2020; Jones and Kording, 2021; Moldwin et al., 2021). Active ion channels are widespread in dendritic arbors, particularly at branch points (nodes) in cortical cells (Borst and Egelhaaf, 1994; Magee, 2008) (Figure 1). In active dendrites, nonlinear summation can occur at every branch point in the dendritic tree, so that dendritic nodes in a single neuron play the computational role (summate and threshold) of entire point neurons in a complex ANN (Figure 1E), essentially granting a single neuron the power of a multi-layer neural network (Poirazi and Mel, 2001; Moldwin and Segev, 2020; Beniaguev et al., 2021; Moldwin et al., 2021).

Active conductances can also play a fundamental role in cell-intrinsic firing patterns, due to coupling between branchlets that leads to intrinsic subthreshold dendritic oscillations (Remme and Torben-Nielsen, 2014). These can also be reflected in firing patterns: Mainen and Sejnowksi (1996) examined cell morphology and showed that dendritic form determines whether the cell fires tonically (roughly periodically) or in concentrated bursts. Thus, the dendritic structure of a cell can strongly influence its firing properties, controlling both the periodicity of firing, and the precise spike timing. Furthermore, as mentioned previously, this dendritic structure is highly plastic: cells are constantly changing their form in an activity-dependent manner, often using the same molecular mechanisms that have previously been researched in a synaptocentric context (Poirazi and Mel, 2001; Lee et al., 2005; Kastellakis et al., 2023). Thus, cell morphology is a crucial intermediate between wetware and electrodynamics.

To illustrate some experimental examples of the computational power of single cells, consider some early results in rodent somatosensation. Rodents flick their whiskers and the resulting sensory signals provide a high-resolution “image” of the space around the head, even in complete darkness. Single cell stimulation studies show that tiny (nano-ampere) currents, applied to single cells, are capable of both generating whisker movement, and eliciting a behavioral response from the animal (as if it had detected a stimulus) (Brecht et al., 2004; Houweling and Brecht, 2008). This is remarkable, given that this low-level stimulation led to only 14 action potentials on average, and that rat somatosensory cortex contains roughly 2 million neurons. This shows that the influence of single cells can be great enough to yield behaviorally detectable consequences, indicating that single-cell computation can have major effects at the whole-brain level (*cf.* Tanke et al., 2018).

To summarize these examples of dendritic computing at the single-cell level, modeling dendrites as simple passive cables already extends the computational powers of dendritic neurons beyond those of point neurons, allowing computations such as motion detection or logical operations like AND to be implemented. But in reality, dendritic trees possess active voltage-gated channels that allow each dendritic branch to spike independently, permitting synaptic inputs to be combined in complex, nested logical fashion. Complex, active dendritic trees thus render each neuron a complex micro-computer in its own right (cf. Poirazi et al., 2003; Cuntz et al., 2014a; Kastellakis et al., 2023). These computations can to some extent be “read off” from the morphology of the dendritic tree, as Cajal had hoped, where complexity of the dendritic tree maps directly onto to complexity of the attendant computation.

Turning to information storage in dendritic trees, the computational discussions above all considered only static neuronal morphology. But ever since Cajal discovered the axonal growth cone, we have known that, like many other eukaryotic cell types, neurons in fact have a highly dynamic form, and can readily change their shape (Van Kerkoerle et al., 2018). This is true of both axonal and dendritic arbors, with axons being distinguished by their long-range “migrations” throughout the brain and body, particularly during development or after injury. New imaging methods have also provided compelling (and beautiful) evidence of plasticity in adult organisms, where filopodia can be observed to extend out from an axon or dendrite, make contact with axons of other neurons, and form new synapses. Rapid morphological changes (particularly in dendritic spines) are correlated with electrical activity of cells and behavioral readouts of whole organisms (Akemann et al., 2010; Roberts et al., 2010; Van Kerkoerle et al., 2018; Kastellakis et al., 2023). All of the computational properties described above depend upon the detailed shape and size of dendrites and/or the distribution of synapses, receptors and ion channels upon the dendritic tree. This means that dynamic changes in dendritic form, and creation or destruction of synaptic contacts, provide an important and capacious potential locus of information storage and cellular memory. For example, changing the location of synapses on the dendritic tree, the overall diameter of a dendrite, or the area of its connection to others at a branch node will all have major effects on its dynamics and coupling to the rest of the cell, thus modifying the overall computation performed by the dendritic tree. This means that, in addition to but independent of synaptic modification, modifications of dendritic form can also serve as a high-capacity locus of learning and memory for individual cells (Poirazi and Mel, 2001).

### Extending artificial neural networks with dendritic computation

As emphasized above, it is no secret that dendritic form plays a central role in cellular neurophysiology, or that active channels exist in dendritic trees: these facts have been suspected since Cajal’s time and clearly documented for at least three decades (for brief histories see Poirazi and Mel, 2001; Shepherd, 2004). This means that single cells are complex microcomputers, whose form fuses computation and information storage in their dendritic morphology (Koch, 1999; Cuntz et al., 2014a; Beniaguev et al., 2021; Kastellakis et al., 2023). Why then do standard contemporary “neural” models ignore this rich domain of neural computation?

It might be expected that including greatly increased computational power in the individual “units” in neural network models would be prohibitively computationally expensive, and beyond the power of existing computers to model. However, recent “deep” neural models of pyramidal cells indicate that more realistic and complex cellular models can, seemingly paradoxically, simplify learning and computation in such networks (Beniaguev et al., 2021; Hodassman et al., 2022). For example, in a network model of a single cell, modifications of synaptic weights during learning can be limited to the superficial input layer (where synapses actually occur in real neurons) and not the deeper layers of the model (corresponding to intra-cellular computation at nodes). Fixing internal node weights corresponds to “freezing” the dendritic structure, allowing most of the neuron’s computation to be modeled in a simple fixed, feed-forward manner (which can be done very efficiently using specialized GPU processing) (Boahen, 2022).

Increased complexity of the “units” in a deep neural network also greatly increases biological plausibility. For example, most contemporary ANNs use back-propagation of error signals throughout an entire network to support learning via synaptic weight adjustment. However, back propagation across many neurons is biologically implausible: there is no known biological mechanism to propagate an error signal across multiple synapses (Roberts, 1989; Lillicrap et al., 2020). In contrast, error signal propagation within a single neuron does exist, due to antidromic propagation of action potentials throughout the dendritic tree, and can serve as a learning signal not only for synapses but for the morphological changes and gene expression changes discussed above as extra-synaptic forms of memory (Schiess et al., 2018). Finally, implementing dendrocentric computation in engineered systems may yield impressive energy savings over the traditional synaptocentric view: Boahen has recently argued that a dendrocentric conception, implemented in silicon, could yield a 400-fold energy savings in engineered “neural networks” (Boahen, 2022). Thus, incorporating insights beyond the synaptocentric standard could yield engineering benefits, while simultaneously making such systems more “neural.”

Thus, more complex models of single neuron computation will allow much closer contact between models of brain function, neural circuits, neuronal form and gene expression, while still allowing robust computational efficiency. Of course, all models must remain constrained to be useful: we cannot simply model every cell in a deep neural network with a full-blown set of partial differential equations, so the search for simplified cell models that nonetheless support more complex computations will be a central desideratum (cf. Boahen, 2022). As our understanding of the computational properties of single neurons increases, varying abstractions regarding their underlying mathematical/computational representation may be required for different purposes (cf. Hedrick and Cox, 2014; Denève and Machens, 2016). But if our goal is to understand how brains compute, there appears to be little justification, either biological or computational, to continue relying upon the outdated point neuron model and the synaptocentric perspective it embodies.

## Discussion: computational and evolutionary implications

In this paper, I have summarized diverse data strongly implying that real neurons are considerably more powerful than “standard model” point neurons, both in terms of computational power and information storage. Although the existence in dendritic arbors of active conductances and nonlinearities has been known for several decades (Koch, 1999; Koch and Segev, 2000), understanding the deeper computational significance of these cellular properties has been a slow process that has only recently reached fruition (Beniaguev et al., 2021; Boahen, 2022; Galakhova et al., 2022; Larkum, 2022; Nandi et al., 2022; Kastellakis et al., 2023). In contrast, understanding the nature of gene regulation and “wetware” has been part and parcel of molecular cell biology almost since its inception (Jacob and Monod, 1961; Bray, 1995), but is too rarely seen in computational terms and integrated into holistic models of single-cell computation in neuroscience. Here, the problem stems from the difficulties of inter-disciplinary integration rather than a dearth of scientific knowledge. Indeed, perhaps the greatest obstacle to synthesizing all of these viewpoints into a unified cognitive perspective on single-cell computation is the sheer volume of knowledge, distributed across different subdisciplines of biology and neuroscience. Having, I hope, demonstrated the possibility and potential promise of such a unified viewpoint, I will end by briefly considering several key implications of the more biologically grounded perspective on neural computation and cognition laid out in this paper.

Beginning with computational implications, dendritic computing in “deep” neurons allows, in a single cell, complex computations previously believed to require multi-layered networks of classical “point” neurons (Poirazi et al., 2003; Moldwin et al., 2021; Larkum, 2022). Larger and more complex dendritic trees can both implement a more complex repertoire of computations, and store more information than point neurons. Single-cell computation provides significantly increased speed and precision (Testa-Silva et al., 2014) at lower energetic cost (Koch, 1999; Niven and Laughlin, 2008; Niven, 2016; Boahen, 2022) than network computation. Thus, changes in both cell morphology and cell-internal factors including intrinsic excitability and wetware will result in highly significant changes in neural computation, both within individual brains and across evolution (summarized in Galakhova et al., 2022).

Summarizing the nature of the computations performed in single cells is a challenge, using currently familiar abstractions like the familiar “analog/digital,” “distributed/symbolic” or “software/hardware” distinctions. Some aspects of dendritic computation are best considered analog (e.g., EPSP propagation in passive dendrites) while others are clearly discrete and digital (e.g., logical operations at active nodes in the dendritic tree, or the cell’s overall binary decision to fire or not). So neurons, and thus neural networks, are mixed analog/digital systems. Similarly, modern silicon-based computers have their wiring diagram fixed during manufacturing (“hardware”), and store information (including software) in flexible and independent memory storage devices of various types (RAM, hard disks, etc.). In contrast, as discussed above, dendritic form influences both the computations the neuron performs and provides a high-capacity, low-cost source of discrete information storage. Because this “hardware” is constantly changing, both influencing computation and storing information, there is no clear hardware/software or CPU/memory distinction in real neurons. Of course, there may be useful related computational abstractions to be made that are more directly relevant to biological computation than those deriving from silicon computing devices. Thus, we urgently need new models of “natural computation” that take into account the biological facts considered in this paper, and should avoid trying to foist existing models of computation, developed mainly in the context of *in silico* computing, onto the biological computers in our skulls.

In particular, the conception of neural computation outlined here calls into sharp question the value of the long running debate regarding discrete, symbolic models of the brain (as in first-generation AI) versus parallel, distributed models (as in late 20th century ANNs or contemporary “deep networks”). Despite the vehemence and persistence of this debate in cognitive science (e.g., Fodor and Pylyshyn, 1988; Elman et al., 1997; Christiansen and Chater, 1999; Marcus, 2001), a serious consideration of cellular neurophysiology shows that artificial neural networks are really no more “neural” than Turing-style symbolic computation. Both perspectives involve oversimplified models whose assumptions, depending on one’s goals, may be more or less appropriate. I suggest that what is needed are new symbolic approaches (cf. Dehaene et al., 2022), perhaps based on the solid mathematical foundations of formal language theory (Fitch and Friederici, 2012; Fitch, 2014), that still allow the high degree of parallelism nicely captured in network models, along with the power and robustness of distributed representations (Rummelhart and McClelland, 1986; Smolensky, 1988). Mathematically, in place of the simple dot product computed by artificial point neurons, a more structured computation that is still tractable in terms of an augmented linear algebra (and computable using modern GPUs) that combines distributed and symbolic computing is clearly desirable and much needed, along the lines of Smolensky’s tensor product proposal (Smolensky, 1990) or Boahen’s “dendrocentric” model (Boahen, 2022).

Turning to cognitive perspectives on brain function, a perspective on neuroscience that centrally includes cellular computation has much to offer, both in terms of synthesizing brain structure and function, and in understanding how genetic changes (over evolution, or among individuals within a species) map onto cognitive function. For example, within vertebrate brains there is a clear gradient of cellular complexity in sensory systems, from numerous simpler cells in primary sensory areas (e.g., V1) to larger and more complex cell structures in higher-order sensory or associations regions (Elston, 2000, 2003; Elston et al., 2009; Galakhova et al., 2022). There is a huge dimensionality expansion in initial stages of cortical computation: human primary visual cortex receives input from roughly 3 million input neurons in the lateral geniculate nucleus, but itself contains about 140 million neurons, implying a 40:1 expansion. Ultimately, however, sensory and motor decisions (e.g., object recognition or action planning) require a great dimensionality reduction to essentially discrete decisions, implying lower numbers of “decision” neurons that sparsely code their outputs (Olshausen and Field, 2004; Houweling and Brecht, 2008). Post-primary processing layers must therefore drastically reduce dimensionality, omitting irrelevant data and compressing representations, in order to converge upon discrete decisions (Tanke et al., 2018). The increased complexity of cells progressing “up” the processing hierarchy suggests that the faster, more precise single-cell computation provided by more complex pyramidal cells in higher-order cortex is one of the mechanisms by which such sparseness and dimensionality reduction are achieved.

Across species, highly significant changes in cell morphology and cell-internal computation have occurred across evolution. These factors almost certainly include both changes in connectivity (particularly long-range connections) and changes in cell-intrinsic computational power (Rilling et al., 2008; Bräuer et al., 2011; Fitch, 2014; Ardesch et al., 2019; Galakhova et al., 2022). For example, numerous studies indicate that cellular complexity, particularly in the dendritic arbor, is higher in humans than in other mammals (Mohan et al., 2015; Galakhova et al., 2022), and that such increased complexity is cognitively relevant (e.g., Ashokan et al., 2018; Goriounova et al., 2018). Dendritic structure is more diverse and varied in human cortex than in macaques or rodents (Mohan et al., 2015; Galakhova et al., 2022), potentially allowing more efficient compression of information at any level of the cortical processing hierarchy. Furthermore, as summarized earlier, increased dendritic complexity allows more computationally distinct dendritic compartments, and thus increased computational power per neuron. This is partially because the lack of synaptic delays and axonal conduction makes single-cell computation faster, more precise, and more energy-efficient (Laughlin et al., 1998; Olshausen and Field, 2004; Niven and Laughlin, 2008; Testa-Silva et al., 2014; Boahen, 2022) than in a network of unstructured point neurons.

Although factors concerning cell shape and connectivity are thus very relevant to cognitive changes across evolution, better understanding how such changes relate to genetics must play a truly central role in understanding the cognitive biology of species differences. Differences in neural form and connectivity are particularly important from the viewpoint of evolutionary genetics. Because single cells are the locus of gene expression, any computational understanding of the rapid evolutionary divergence in neurally-expressed genes (cf. Theofanopoulou et al., 2017) will require an increased understanding of how differences in gene expression map onto changes in the morphology and connectivity of single neurons. This is true both within a species (e.g., to understand individual variability and clinical disorders, Glessner et al., 2009; Goriounova et al., 2018) and across species (DeFelipe, 2011; Galakhova et al., 2022).

Recent advances in transcriptomics reveal important changes in gene expression in human cortical cells relative to those of rodents. Based on expression of key genes, humans have unique pyramidal cell types not seen in rodents. For example, some human pyramidal cells express CARM1P1 or FREM3 which code for neurofilament markers indicative of long-range cortico-cortical connections (Berg et al., 2021). These transcription factor differences reflect robust differences in both cell morphology and electrical properties (Nandi et al., 2022), such as presence and timing of dendritic spikes, and in some cases these differences have already been shown to increase the computational power of such cells (Gidon et al., 2020). Thus, changes in gene expression thought to be cognitively relevant will play out first and foremost at the level of single-cell morphology and development – whether at the cell-structural level, the distribution of receptors and ion channels within the cell, or the connections between cells. Given the practical and theoretical relevance of understanding the mapping between genes, brains and minds, cellular computation should thus take center stage in the next generation of cognitive and computational models of the brain.

## Summary and conclusions

My central argument in this paper has been that every eukaryotic cell is a complex computer at the levels of gene expression and “wetware” (Bray, 2009; Brenner, 2012; Fitch, 2021), and that neurons in particular add additional layers of computation to these in their dendritic form and cell-to-cell connectivity (Koch and Segev, 2000; Beniaguev et al., 2021). By analogy with contemporary “deep” neural networks, “deep” neurons constitute powerful microcomputers at the cellular level. However, these computations occur at multiple different levels and time scales, ranging from very rapid wetware and electrodynamics to the much slower formation of long-range neural connections during development. The synaptocentric view of most modern neural networks, in contrast, pictures neurons as simple sum-and-threshold nodes, where all of the computational work is done at the network level, and information storage occurs solely in synapses via adjustable synaptic weights.

Although I have distinguished four different computational substrates in this review, each of these four levels interacts with the others. Although this perspective may seem very (perhaps unnecessarily) complicated to an engineer, it is first of all the way biology does “natural computation” (as a matter of fact) and (as a matter of principle) it allows an integration of the explanatory levels of genetics, biochemistry, cell form and neural circuitry in a way inaccessible to standard “neural” models. Thus, if we hope to understand how genetic changes during evolution impinge upon neural circuitry, and thus control brain computation and cognition, we must embrace a cell-focused viewpoint on computation, along with the complexity that attends it, and not continue to focus solely on network structure.

The viewpoint on cellular computation advanced here has an important implication for the arguments of Gallistel and colleagues (Gallistel and King, 2010; Gallistel, 2020; Langille and Gallistel, 2020), who have argued that neural network models (and thus various conceptions of neural function that are based on them) are intrinsically unsuited to provide satisfactory models of cognition and memory due to their fundamental reliance on stored associations (cf. Dayan, 2009; Gershman et al., 2021; Prasada, 2021; Poeppel and Idsardi, 2022). My arguments here are consistent with Gallistel’s critique, insofar as I argue that synaptic weights are not the sole repository of long-term stored information in the brain. However, I have tried to show here that there are multiple well-studied domains of information storage that *can* play this role, and thus that we need not rely on any novel undiscovered mechanisms (e.g., reverse transcription of learned information into DNA Gallistel, 2020) to fill the explanatory gaps left by rejecting a synaptocentric view. I argue that all we need to solve “Gallistel’s problem” is to take seriously the known molecular biology of cells in general, along with the computational properties embodied in the form of neurons in particular. When we do so, we discover a surfeit of possible information storage mechanisms at the level of single cells that are discrete, long-lasting and metabolically inexpensive: precisely Gallistel’s desiderata. The task moving forward will be to better integrate our understanding of these levels, and to better understand how these different mechanisms, each of them a topic of a discipline in its own right, interact to provide the computational and information storage resources that underlie cognition in humans and other animals.

## Data availability statement

The original contributions presented in the study are included in the article/supplementary material, further inquiries can be directed to the corresponding author.

## Author contributions

WTF conceived and wrote the article.
